# Effect of Intensity on Changes in Cardiac Autonomic Control of Heart Rate and Arterial Stiffness After Equated Continuous Running Training Programs

**DOI:** 10.3389/fphys.2021.758299

**Published:** 2021-12-09

**Authors:** Mohammad Soltani, Masoud Jokar Baluchi, Daniel Boullosa, Ali Daraei, Patricia K. Doyle-Baker, Ayoub Saeidi, Beat Knechtle, Kambiz Moradi Dehbaghi, Shirin Shirzad Mollabashi, Trisha A. VanDusseldorp, Hassane Zouhal

**Affiliations:** ^1^Department of Biological Sciences in Sport, Faculty of Sports Sciences and Health, Shahid Beheshti University, Tehran, Iran; ^2^Department of Exercise Physiology, Faculty of Physical Education and Sports Sciences, Kharazmi University, Tehran, Iran; ^3^Integrated Institute of Health, Federal University of Mato Grosso do Sul, Campo Grande, Brazil; ^4^Sport and Exercise Science, James Cook University, Townsville, QLD, Australia; ^5^School of Health and Exercise Sciences, The University of British Columbia, Kelowna, BC, Canada; ^6^Faculty of Kinesiology, University of Calgary, Calgary, AB, Canada; ^7^Department of Physical Education and Sport Sciences, University of Kurdistan, Sanandaj, Iran; ^8^Medbase St. Gallen Am Vadianplatz, St. Gallen, Switzerland; ^9^Institute of Primary Care, University of Zūrich, Zurich, Switzerland; ^10^Department of Physical Education, Islamic Azad University, Tehran, Iran; ^11^Faculty of Sport Sciences, Atatürk University, Erzurum, Turkey; ^12^Department of Exercise Science and Sport Management, Kennesaw State University, Kennesaw, GA, United States; ^13^Laboratoire Mouvement, Sport, Santé – EA 1274, University of Rennes, Rennes, France; ^14^Institut International des Sciences du Sport (2I2S), Irodouer, France

**Keywords:** autonomic function, HRV, vascular function, arterial stiffness, training intensity, blood pressure

## Abstract

**Background:** It is well known that exercise training has positive effects on both cardiac autonomic function and arterial stiffness (AS). However, it is not clear that which exercise training variables, intensity or volume, or both, play a crucial role in this regard. This study investigates the chronic effects of high-volume moderate-intensity training (HVMIT) and low-volume high-intensity training (LVHIT) on heart rate variability (HRV) and AS in sedentary adult men.

**Materials and Methods:** Notably, 45 males (age: 42 ± 5.7 years) were randomly assigned to a control (*n* = 15), HVMIT (*n* = 15), or LVHIT (*n* = 15). The HVMIT group ran three times per week on a treadmill at 50–60% of VO_2_max for 45–60 min, while the LVHIT trained at 70–85% of VO_2_max for 25–40 min. Both training protocols were equated by caloric expenditure. HRV, pulse wave velocity (PWV), hemodynamic variables, and body composition were measured before and after 12 weeks.

**Results:** Both protocols (i.e., HVMIT and LVHIT) significantly increased the SD of normal sinus beat intervals (SDNN) and high-frequency (HF) bands (*p* < 0.05) after 12 weeks. Whereas the low-frequency (LF)-HF ratio decreased significantly in both training protocols (*p* < 0.05); however, these changes were significantly greater in the LVHIT protocol (*p* < 0.05). Furthermore, the root mean square of successive RR interval differences (RMSSD) significantly increased only in the LVHIT (*p* < 0.05). Moreover, a significant decrease in LF and PWV was only observed following the LVHIT protocol (*p* < 0.05). Some measures of HRV and PWV were significantly correlated (*r* = 0.275–0.559; *p* < 0.05).

**Conclusion:** These results show that the LVHIT protocol was more efficient for improving HRV variables and PWV than the HVMIT protocol after 12 weeks of continuous running training. Interestingly, changes in some HRV parameters were related to changes in PWV. Further studies should elaborate on the link between central and peripheral cardiovascular adaptations after continuous and intermittent training regimens differing in intensity.

## Introduction

Physical inactivity and sedentary behavior are key lifestyle-related risk factors for developing cardiovascular diseases (CVD) and are associated with an increased risk of all-cause mortality and morbidity (Lavie et al., 2019). The cardiac autonomic function is a variable affected by a sedentary lifestyle, and the impairment of its function is a predictable marker for CVD (Lavie et al., 2019; Niemelä et al., 2019). Heart rate variability (HRV) is a noninvasive method for evaluating the cardiac autonomic control of heart rate (HR), which represents electrical cardiac variation by the time interval between R-R intervals (Thayer et al., 2010). HRV is tightly controlled by the autonomic nervous system (ANS) through parasympathetic modulations (Thayer et al., 2010). Previous studies indicate that the decrease in HRV (i.e., reduced vagal modulations) is associated with an increased risk of CVD, sudden cardiac death, and all-cause mortality (Thayer et al., 2010; Sessa et al., 2018). Recently, HRV has been suggested as a method to monitor the influence of sedentary behavior on the cardiac autonomic control of HR (Niemelä et al., 2019).

Arterial stiffness (AS) is typically measured by pulse wave velocity (PWV) and is a marker of vascular function as well as an independent risk factor for cardiovascular events and overall mortality (Mattace-Raso et al., 2006; Mitchell, 2009). The specific role that the ANS plays with respect to AS is somewhat contentious (Mäki-Petäjä et al., 2016). However, the ANS has a regulatory role in HR and vascular tone and therefore might augment AS (Mäki-Petäjä et al., 2016; Sheng and Zhu, 2018). A sedentary lifestyle may be accompanied by a dysfunction of the ANS (i.e., decrease in HRV) and impairment of vascular function (i.e., increase in PWV), thus leading to an increased risk of CVD (Thayer et al., 2010; Ahmadi-Abhari et al., 2017).

Regular exercise training could be an effective way to counteract the above-mentioned sedentary lifestyle consequences on cardiovascular function (Ahmadi-Abhari et al., 2017). Exercise training accompanied by improved cardiorespiratory fitness increases vagal modulations, which plays an important role in improving HRV (Locateli et al., 2018; Singh et al., 2018; Veijalainen et al., 2019). Furthermore, exercise training improves vascular function and decreases AS by promoting shear stress and nitric oxide (NO) bioavailability, thereby reducing oxidative stress and inflammation (Birk et al., 2013; Cocks et al., 2016; Lessiani et al., 2016). Although the effects of exercise training on either HRV or AS have been determined, it remains unclear that which exercise training variables, intensity or volume, are associated with greater adaptations (Dos Santos et al., 2019). Previous studies have demonstrated that similar positive improvements of HRV and vascular function follow both moderate-intensity and high-volume exercise training in comparison to high-intensity exercise training (Bhati et al., 2017; Rodrigues et al., 2020). In contrast, other studies show greater improvements in HRV and AS following high-intensity training (Bahmanbeglou et al., 2019; Ramírez-Vélez et al., 2020). These contradictory results may be due to differences between the exercise mode and workloads, as well as the differences in the training duration, sex, age, and nutritional status of participants, and training status (sedentary vs. trained) (Medeiros et al., 2021). Moreover, the workloads of most of the previous studies were not equated internally (caloric expenditure) (Kilpatrick et al., 2009; Alansare et al., 2018) or externally (Km per week) (Alansare et al., 2018; Ramírez-Vélez et al., 2020), which make it challenging to compare protocols with different intensities and volumes. In addition, previous studies have not reported adherence to their protocols, which could have an important clinical impact on designing exercise protocols for sedentary people (Alansare et al., 2018; Ramírez-Vélez et al., 2020).

Therefore, the purpose of this study was to investigate the effects of high-volume moderate-intensity (HVMIT) and low-volume high-intensity training (LVHIT) with equated caloric expenditure and workloads (Km/per week) on HRV and AS in sedentary young men. We hypothesized that LVHIT would improve HRV and decrease AS when compared to HVMIT.

## Materials and Methods

### Study Population

A total of 45 sedentary men were recruited *via* advertisements which included posters, emails, and social media platforms (see Table 1 for participant characteristics). The inclusion criteria were as follows: (1) had not participated in regular physical activity in the last 6 months, (2) not meeting the exercise recommendations of the American College of Sports Medicine (150 min moderate-intensity exercise or 75 min vigorous exercise per week), (3) non-smokers, (4) free of a history of chronic diseases particularly CVD, and (5) not taking any medication over the last 6 months. Participants were excluded if they had joint or skeletal muscle disorders and if they were using ergogenic aid supplements. An experienced clinical exercise physiologist screened participants for normal resting blood pressure (BP) and HR (RHR). The informed consent was completed by participants prior to the start of the study which described the experimental procedures, risk, and potential benefits associated with the study. The study procedures were reviewed and approved by the local University Research and Ethics Committee (Ethics code: IR-IAU1398-18). All procedures were performed according to the latest revision of the Declaration of Helsinki.

**TABLE 1:** Mean and SD (±) characteristics of participants.

Characteristics	Control	HVMIT	LVHIT
Age (year)	41.5 ± 5.6	42.5 ± 6.2	42.2 ± 5.3
Height (cm)	175.3 ± 4.2	174.8 ± 3.6	177.6 ± 4.3
Body mass (kg)	74.9 ± 6.8	73.5 ± 5.5	73.4 ± 6.0
BMI (kg/m^2^)	24.4 ± 1.4	24.0 ± 1.3	23.3 ± 1.5
BF (%)	29.5 ± 5.1	27.5 ± 3.0	28.5 ± 4.0
FFM (Kg)	24.5 ± 2.6	25.3 ± 3.2	25.5 ± 3.3
SBP (mmHg)	128.1 ± 2.1	128.2 ± 1.6	129.4 ± 2.0
DBP (mmHg)	75.3 ± 7.9	73.2 ± 6.7	74.1 ± 7.0
VO_2_max (ml/kg/min)	33.5 ± 5.0	33.3 ± 4.5	35.2 ± 4.1

*HVMIT, high-volume moderate-intensity training; LVHIT, low-volume high-intensity training; BMI, body mass index; BF, body fat percentage; FFM, fat-free mass; SBP, systolic blood pressure; DBP, diastolic blood pressure.*

### Experimental Design

**FIGURE 1:**
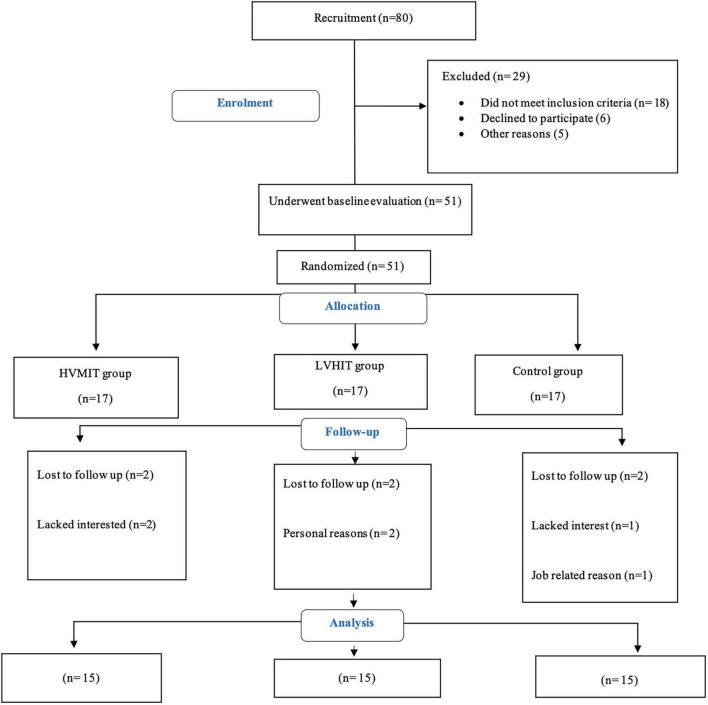
Participants flow chart. HVMIT, high-volume moderate-intensity training; LVHIT, low-volume high-intensity training.

Notably, 15 participants were randomly allocated into three groups (i.e., HVMIT, LVHIT, or control) using Random Allocation Software (Isfahan, Iran) (Figure 1). The following biometrics, i.e., resting HRV, PWV, resting BP, body composition, and VO_2_max, were assessed 48 h before the start and 48 h after the last exercise session of both training protocols. Instructions were given to refrain from (1) consuming any caffeine-contained beverages and (2) participating in moderate to vigorous physical activity 24 h prior to the measurements. All measurements were conducted in the morning under the same environmental conditions (∼20°C and ∼55% relative air humidity).

### Heart Rate Variability

Participants arrived at the laboratory between 8:00 a.m. to 10:00 a.m. in a fasting state (12 h). Before completing the HRV monitoring, participants were seated in a quiet room with the lights off and the temperature was controlled between 22 and 23°C. All procedures were performed according to the standards proposed by the Task Force of the European Society of Cardiology (Marek, 1996). The RR recordings were captured by an HR monitor (Polar Electro, Kempele, Finland). The resting HRV was evaluated for 25 min in the supine position with the first 5 min devoted to signal stabilization which was not included in the analyses. Normal breathing frequency and tidal volume were maintained during the testing. All artifact and ectopic data were filtered and corrected with a minimum protection zone of six beats. Any recording data with more than 5% error was discarded. A computer software program (The Biomedical Signal and Medical Imaging Analysis Group, Department of Applied Physics, University of Kuopio, Finland) was used to analyze both time and frequency domain data (Tarvainen et al., 2014). The time domain of HRV included the SD of RR intervals (SDNN), index of overall variability, and root mean square of successive differences between RR intervals (RMSSD) as the measure associated with vagal modulations. The frequency-domain parameters included the low-frequency (LF: 0.04–0.15 Hz) and high-frequency (HF: 0.15–0.40 Hz) bands, and the analysis of power spectrum was calculated by using fast Fourier transformation (FFT; welch, 256 points Hanning-windowing, Kubios HRV Analysis, Biosignal Analysis, and Medical Imaging Group, University of Eastern Finland, Finland). The ratio of the LF to HF (LF-HF) bands was also evaluated. For time-domain variables and frequency domain parameters, the reproducibility of 24-h-derived HRV indices was very good and excellent with ICC values of 0.85 and 0.90, respectively.

### Resting Blood Pressure and Pulse Wave Velocity

Blood pressure (SBP/DBP) was measured according to the guidelines of the European Society of Hypertension (O’Brien et al., 2005). Before the measurement of BP, participants were seated on a comfortable chair for 10 min and an electronic sphygmomanometer (BPM AM 300P CE, Kenz, Suzuken Company, Japan) was used. This was followed by the HRV measurements. PWV (AS index) was measured with a vascular explorer (Enverdis, Jena, Germany) in a supine position after resting for 10 min. Analysis of PWV and BP were performed by photoplethysmography sensors and inflatable upper arm and lower leg cuffs techniques, as described elsewhere (Nürnberger et al., 2011). The reproducibility of PWV was very good with an ICC value of 0.86.

### Anthropometry and Body Composition

Body weights (to the nearest 0.1 kg, Seca, Birmingham, United Kingdom) and heights (to the nearest 0.1 cm, Seca, Birmingham, United Kingdom) were evaluated (light clothing and no footwear after an overnight fast) by a digital scale and a stadiometer. The body mass index (BMI) was calculated by dividing body weight (kg) by the square of their height (m^2^). The bio-impedance analyzer (X-Scan Plus 950, Medigate Company Inc., Dan-dong Gunpo, South Korea) was used to measure fat-free mass (FFM) and body fat percentage (BF%).

### Cardiorespiratory Fitness

A maximal oxygen uptake (VO_2_max) test was conducted on a motorized treadmill (H/P/Cosmos, Pulsar med 3p Sports and Medical, Nussdorf-Traunstein, Germany) with a start speed of 6 km⋅h^–1^. The treadmill grade was set and maintained at 0.5° while the speed increased by 1 km⋅h^–1^ every 3 min until participants were either physically exhausted or unable to continue. A gas analyzer system (Metalyzer 3B analyzer, CORTEX Biophysik GmbH, Germany) was used to measure oxygen uptake (VO_2_) and was calibrated before each test based on the instructions of the manufacturer. The highest 60-s VO_2_ value during the test was considered the VO_2_max. The last completed 3-min stage was identified as the maximal testing velocity (Vmax). Details on the VO_2_max test protocol have been previously described (Nummela et al., 2016).

### Nutrient Intake and Dietary Analysis

Participants were instructed to document all food intakes as accurately as possible over 3 days, weekly (2 weekdays and 1 day on the weekend), throughout the study period. The weekly dietary records were assessed using Diet Analysis Plus, version 10 (Cengage, Boston, MA, United States), and total energy (kcal per day), carbohydrate, fat, and protein (grams per day) intakes were calculated.

### Running Training Protocols

Both groups were supervised during the three exercise sessions per week for the entire 12 weeks. The HVMIT protocol was progressive with the first 2 weeks at 50% of VO_2_max for 45 min, followed by 6 weeks (3–7) at 55% of VO_2_max for 50 min, and with the final 5 weeks (8–12) at 60% of VO_2_max for a 60-min duration. Similarly, the LVHIT protocol was progressive, but the first 2 weeks were at 70% of VO_2_max for 25 min, followed by 6 weeks (3–7) at 80% of VO_2_max for 35 min, and with the final 5 weeks (8–12) at 85% of VO_2_max for a 40-min duration. Caloric expenditure for both protocols was calculated to be the same (14 kcal/kg of body mass per week), and the amount of distance run per week was also similar between protocols (17.5 km). The intensity in both protocols was controlled and initially set by Vmax. An HR monitor (Polar V800, Polar Electro Oy, Finland) was used in all sessions to record HR. Adherence of participants to the protocol was recorded and calculated by dividing the number of minutes completed within the identified HR range each week by the minutes prescribed (Table 2). The VO_2_max test was repeated at weeks 4 and 8 to ensure that the target intensity was maintained given that some participants likely benefited from the training and improved. The control group was asked to maintain their daily routines and refrain from any additional physical activity for the duration of the study. See Table 2 for exercise protocols.

**TABLE 2:** Workload prescription and adherence to both training protocols.

Variables	HVMIT	LVHIT
Mean Intensity (%VO_2_max)	56%	80%
Prescription amount (km/wk)	17.5	17.5
Prescription amount (kcal-kg-wk)	14	14
Prescription time (min/wk)	160	106
Adherence %	95 (6%)	92 (8%)
Actual amount (km/wk)*	14.25 ± 8.3	13.8 ± 7.6
Actual time (min/wk)**	156 ± 5.1	92 ± 4.2
Frequency (sessions/wk)	3	3

*HVMIT, high-volume moderate-intensity training; LVHIT, low-volume high-intensity training; Vmax, maximal velocity; wk, week.*

*Actual amount: Prescription amount × adherence.

**Actual time: Prescription time × adherence.

### Statistical Analysis

All data were described by means and SD (±). The normality of all data was evaluated using the Shapiro–Wilk test. A one-way ANOVA was applied to compare all baseline data among the three groups to ensure no significant differences between groups. A two-way ANOVA with repeated measures (groups by time) was used to compare the changes of three groups for all variables. A Bonferroni *post hoc* test was run following a significant group-by-time interaction. Correlation between changes in PWV and HRV variables before and after training was measured using the Pearson product-moment correlation test. Additionally, the effect sizes (ES) were determined from the ANOVA output by partial eta-squared. Within-group ES were computed using the following equation: ES = (mean post - mean pre)/SD (Field, 2013). In accordance with Hopkins et al. (2009), ES were considered trivial (<0.2), small (0.2–0.6), moderate (0.6–1.2), large (1.2–2.0), and very large (2.0–4.0). The level of significance was set at *p* < 0.05. All statistical analyses were computed using SPSS for Windows, version 23.0 (SPSS Inc., Chicago, United States). The sample size was designed to detect a difference in study variables with a 95% CI and ≥80% power value using G power software.

## Results

In all study groups (i.e., control, HVMIT, and LVHIT), no significant differences (*p* > 0.05) were observed for age, weight, BMI, BF%, FFM, SBP, DBP, and VO_2_max (Table 1). Moreover, there were no significant differences (*p* > 0.05) between the groups for HRV variables and PWV at baseline (Table 3).

**TABLE 3:** Pretest and the posttest mean and SD (±) values of HRV indices, hemodynamic variables, VO_2_max, Vmax, and body composition variables.

Variable	Group	Pre	Post	Partial Eta Squared	CV (%)
SDNN (m/s)	HVMIT	65.4 ± 7.3	74.0 ± 6.0^‡^^*^	0.49	13
	LVHIT	65.5 ± 7.7	84.6 ± 8.0^‡^^*^^#^		29
	Control	62.4 ± 5.2	60.3 ± 5.8		−3
RMSSD (m/s)	HVMIT	70.2 ± 7.3	77.4 ± 7.3^‡^	0.85	10
	LVHIT	72.3 ± 7.9	88.1 ± 8.2^‡^*		22
	Control	68.2 ± 7.6	69.3 ± 7.4		2
HR (bpm)	HVMIT	79.2 ± 7.6	70.4 ± 7.6^‡^	0.59	−11
	LVHIT	80.1 ± 7.7	68.4 ± 7.7^‡^		−15
	Control	79.5 ± 7.7	78.3 ± 5.4		−2
LF (ms^2^)	HVMIT	1751.2 ± 116.3	1625.3 ± 131.3^‡^	0.95	−7
	LVHIT	1784.3 ± 116.1	1323.3 ± 110.4^‡^^*^^#^		−26
	Control	1650.5 ± 116.3	1690.4 ± 118.3		2
HF (ms^2^)	HVMIT	1925.4 ± 117.1	2322.2 ± 161.3^‡^*	0.92	21
	LVHIT	1875.1 ± 115.2	2652.1 ± 172.3^‡^^*^^#^		41
	Control	2015.1 ± 116.3	1992.4 ± 100.5		−1
LF/HF	HVMIT	0.90 ± 0.01	0.70 ± 0.03^‡^*	0.98	−22
	LVHIT	0.95 ± 0.00	0.50 ± 0.02^‡^^*^^#^		−47
	Control	0.81 ± 0.01	0.84 ± 0.02		4
VO_2_max (mL/kg/min)	HVMIT	33.3 ± 4.5	36.1 ± 4.4^‡^	0.76	8
	LVHIT	35.2 ± 4.1	40.7 ± 3.6^‡^*		16
	Control	33.5 ± 5.0	33.8 ± 4.4		1
V_max_ (km/h)	HVMIT	12.0 ± 1.4	12.8 ± 1.5^‡^	0.64	7
	LVHIT	12.5 ± 1.7	14.3 ± 1.7^‡^		14
	Control	12.3 ± 1.7	12.0 ± 1.6		−2
Body mass (kg)	HVMIT	73.5 ± 5.5	71.2 ± 5.7	0.76	−3
	LVHIT	73.4 ± 6.0	70.5 ± 6.6		−4
	Control	74.9 ± 6.8	75.1 ± 5.6		0
BMI (kg/m^2^)	HVMIT	24.0 ± 1.3	23.3 ± 1.6	0.55	−3
	LVHIT	23.3 ± 1.5	22.3 ± 1.8		−4
	Control	24.4 ± 1.4	24.4 ± 1.5		0
BF (%)	HVMIT	27.5 ± 3.0	26.5 ± 2.9^‡^	0.76	−4
	LVHIT	28.5 ± 4.0	23.6 ± 3.1^‡^*		−17
	Control	29.5 ± 5.1	30.2 ± 5.1		2
FFM (kg)	HVMIT	25.3 ± 3.2	25.9 ± 3.1	0.22	2
	LVHIT	25.5 ± 3.3	26.5 ± 3.7		4
	Control	24.5 ± 2.6	24.1 ± 2.5		−2

*SDNN, SD of RR intervals; RMSSD, root mean square successive difference of RR intervals; HF, high frequency; LF, low frequency; Vmax, maximum velocity; BMI, body mass index; BF, body fat percentage; FFM, fat-free mass; CV, change value; HVMIT, high-volume moderate-intensity training; LVHIT, low-volume high-intensity training.*

*Significant difference with the control group (p < 0.05).

*^#^Significant difference between training protocols (p < 0.05).*

*^‡^Indicates significant difference from baseline (p < 0.05).*

**FIGURE 2:**
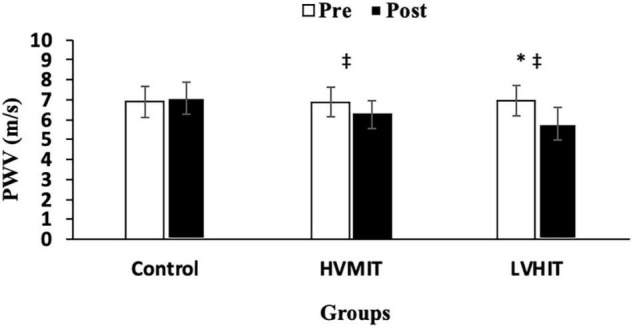
Pretraining and posttraining values [mean, SD (±)] for pulse wave velocity (PWV). HVMIT, high-volume moderate-intensity training; LVHIT, low-volume high-intensity training. *Indicates significant differences from the control group (*p* < 0.05). ^‡^Indicates significant difference from baseline (*p* < 0.05).

**FIGURE 3:**
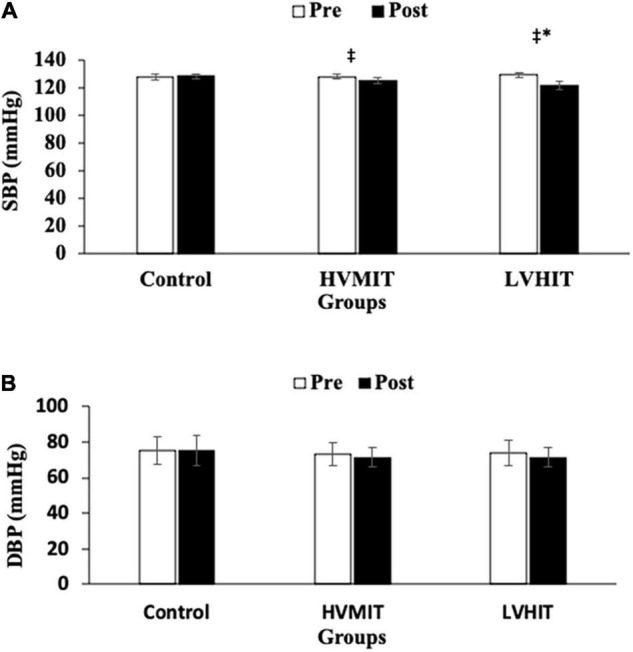
**(A)** Pretraining and posttraining values [mean, SD (±)] for systolic blood pressure (SBP). **(B)** Pretraining and posttraining values [mean, SD (±)] for diastolic blood pressure (DBP). HVMIT, high-volume moderate-intensity training; LVHIT, low-volume high-intensity training. *Indicates significant differences from the control group (difference between training protocols with the control group) (*p* < 0.05). ^‡^Indicates significant difference from baseline (*p* < 0.05).

The following results can be found in Table 3, unless otherwise stated. A significant interaction between group and time was observed for SDNN (*F*2,42 = 20.9 and *p* < 0.05), RMSSD (*F*2,42 = 124 and *p* < 0.05), LF (*F*2,42 = 403 and *p* < 0.05), HF (*F*2,42 = 263 and *p* < 0.05), LF-HF (*F*2,42 = 1,043 and *p* < 0.05), HR (*F*2,42 = 30.6 and *p* < 0.05), PWV (*F*2,42 = 286 and *p* < 0.05), SBP (*F*2,42 = 71.4 and *p* < 0.05), BF% (*F*2,42 = 69.3 and *p* < 0.05), VO_2_max (*F*2,42 = 67.5 and *p* < 0.05), and Vmax (*F*2,42 = 38.4 and *p* < 0.05). SDNN and HF significantly increased in both training protocols (i.e., HVMIT and LVHIT), when compared with the control group (*p* < 0.05). However, these increases were significantly greater in LVHIT when compared with the HVMIT protocol (*p* < 0.05). Moreover, paired sample *t*-test indicated significant increases (*p* < 0.05) in SDNN and HF following 12 weeks when compared with baseline in both training protocols. Data analysis showed a significant decrease in LF-HF ratio in HVMIT and LVHIT protocols in comparison with the control group (*p* < 0.05). However, a more significant decrease was observed in LVHIT compared with the HVMIT protocol (*p* < 0.05). Both training protocols showed significant decreases (*p* < 0.05) in LF-HF ratio post 12 weeks when compared with the baseline. Furthermore, RMSSD (increased) and LF (decreased) showed significant changes only in the LVHIT protocol when compared with control group (*p* < 0.05). However, these changes in the HVMIT protocol were not significant (*p* > 0.05). The changes from baseline to 12 weeks in RMSSD and LF were significant (*p* < 0.05) in HVMIT and LVHIT protocols. HR showed no significant difference (*p* > 0.05) in either training protocols compared with the control group. But a significant reduction (*p* < 0.05) in both training protocols from baseline to 12 weeks was demonstrated. PWV (ES: 0.93), SBP (ES: 0.77), and BF% significantly decreased in LVHIT compared with the control group (*p* < 0.05) (Figures 2, 3 and Table 3). However, no significant changes were observed in the HVMIT protocol (Figures 2, 3 and Table 3).

There were significant correlations in both training conditions (i.e., LVHIT and HVMIT) (*p* < 0.05) between PWV with SDNN (*r* = −0.524), RMSSD (*r* = −0.358), HR (*r* = 0.276), LF (*r* = 0.442), HF (*r* = −0.481), and LF-HF (*r* = 0.559) as determined by the Pearson correlation analyses (Table 4).

**TABLE 4:** Pearson correlation analysis of associations between PWV with SDNN, RMSSD, HR, LF, HF, and LF-HF.

		Biomarkers	Pearson *r*-value	*p*-value
LVHIT	PWV (m/s)	SDNN (m/s)	−0.652	*p* < 0.05*
		RMSSD (m/s)	−0.525	*p* < 0.05*
		HR (bpm)	0.298	*p* < 0.05*
		LF (ms^2^)	0.440	*p* < 0.05*
		HF (ms^2^)	−0.480	*p* < 0.05*
		LF/HF	0.589	*p* < 0.05*
HVMIT	PWV (m/s)	SDNN (m/s)	−0.425	*p* < 0.05*
		RMSSD (m/s)	−0.351	*p* < 0.05*
		HR (bpm)	0.252	*p* < 0.05*
		LF (ms^2^)	0.369	*p* < 0.05*
		HF (ms^2^)	−0.385	*p* < 0.05*
		LF/HF	0.424	*p* < 0.05*

*SDNN, SD of RR intervals; RMSSD, root mean square successive difference of RR intervals; HF, high frequency; LF, low frequency; LVHIT, low-volume high-intensity training; HVMIT, high-volume moderate-intensity training.*

*Indicates the significant correlation between levels of the measured variable with pulse wave velocity (PWV) levels.

Neither training protocol showed significant decreases from baseline to 12 weeks in PWV, SBP, and BF%. VO2max significantly increased only in the LVHIT protocol when compared with the control group (*p* < 0.05). However, both training protocols resulted in a significant increase (*p* < 0.05) in VO2max from baseline to 12 weeks. Vmax changes in both training protocols were not significant when compared with the control group. However, these changes in HVMIT and LVHIT were significant (*p* < 0.05) after 12 weeks when compared with baseline. No significant changes were observed in DBP, BMI, and FFM in both training protocols when compared with the control group (Figure 3 and Table 3).

## Discussion

This study examines the exercise effects of different volumes and intensities of endurance training on HRV variables, PWV, cardiorespiratory fitness, hemodynamic markers, and body composition in sedentary adult males. The main findings were that the LVHIT is more effective in improving HRV variables (i.e., SDNN, RMSSD, LF, HF, and LF-HF), PWV SBP, VO_2_max, Vmax, and BF% when compared with an HVMIT protocol. Thus, a shorter protocol of higher intensity was more effective for improving cardiovascular health metrics in sedentary adult men.

Both training protocols improved the time domain (SDNN and HR) and frequency domain (HF and LF/HF) variables; however, this improvement was more significant in the LVHIT than the HVMIT protocol. Moreover, RMSSD and LF showed improvement by only following the LVHIT protocol. This improvement of HRV agrees with other studies, which show that exercise intensity plays a crucial role in modifying HRV variables (Heydari et al., 2013; Alansare et al., 2018). It is well established that cardiovascular adaptation resulting from exercise training can be affected by the ANS (Michelini and Stern, 2009; Fürholz et al., 2013). Exercise training can decrease sympathetic activity and increase parasympathetic activity, thus decreasing resting HR, which was observed to positively change early on during the 12 weeks of exercise (Rosenwinkel et al., 2001). Moreover, a greater increase in epinephrine and norepinephrine has been observed when exercise is performed with higher intensities, suggesting a higher sympathetic nervous system activity and greater physiological stress during exercise sessions (Jacob et al., 2004). This might lead to greater physiological and cardio-autonomic adaptations in the short term (Aubert et al., 2003; Jacob et al., 2004). Furthermore, an increase in catecholamine concentration could increase lipolysis in adipose tissues, which may be why there was a significant reduction in BF% following the LVHIT protocol (Zouhal et al., 2013). Exercise intensity is also known to increase shear stress and subsequently plays a critical role in releasing NO in the arteries (Cocks et al., 2016). NO could be responsible for modulating the cardio-autonomic system by decreasing sympathetic outflow and increasing parasympathetic influences (Schultz, 2009). Improvements in the variables associated with HRV in the LVHIT protocol might be explained by these physiological adaptations that occur following shorter duration and higher intensity endurance exercise training.

The PWV and SBP also decreased significantly after 12 weeks in the LVHIT but not in the HVMIT protocol. This is somewhat surprising given that previous studies have reported improvement in AS and SBP following high-intensity exercise training (Bahmanbeglou et al., 2019; Soltani et al., 2020). Previous studies have also observed that high-intensity exercise when compared with low or moderate exercise has greater effects on vascular function. This occurs through enhanced shear stress and plasma NO production, with a subsequent increase in vasorelaxation (Ramos et al., 2015; Cocks et al., 2016). We speculated that the LVHIT would have more significant effects than the HVMIT protocol on vascular function due to greater stimulation of the shear rate resulting in increasing NO bioavailability and as a result, improved PWV and SBP. Furthermore, improvement of the arterial baroreflex sensitivity following exercise training plays a significant role in modulating HR and vascular tone and therefore could be associated with reducing AS and increasing cardiac autonomic function (Heydari et al., 2013; Chaswal et al., 2015; Sheng and Zhu, 2018). In addition, other mechanisms, such as a decrease in inflammation and oxidative stress, may have a contributing role in reducing PWV (Munk et al., 2011; Bogdanis et al., 2013; Ismaeel et al., 2018).

The significant increase in VO_2_max only in the LVHIT protocol is another interesting finding in our study and supports the results found by Ross et al. (2015). These authors reported a greater increase in VO_2_max following continuous training at 75% relative to 50% of VO_2_max (Ross et al., 2015). This increase was attributed to both central (i.e., cardiac output increase) and peripheral (i.e., mitochondrial density) adaptations (MacInnis and Gibala, 2017). The improvement in PWV may also be related to central adaptations following the LVHIT protocol (Boreham et al., 2004). Improvements in VO_2_max, SBP, and PWV reinforce the importance of exercise intensity for inducing physiological adaptation, which leads to improvement in HRV variables. Röhling et al. (2017) suggested that it is possible that an increase in VO_2_max would result in an improvement in cardiac autonomic function which would have a significant effect on parasympathetic modulations (Ueno et al., 2002). Furthermore, one novel finding in this study is the low-to-moderate correlations found between HRV parameters and PWV, which would suggest some interplay between central and peripheral cardiovascular mechanisms after training that warrants further research.

The limitation of this study is that we did not measure the NO or baroreceptor sensitivity, and the inclusion of these measurements may have increased our understanding of the mechanisms behind some of the findings. The reliability and validity of BIA have been confirmed with previous studies (Jackson et al., 1988; Ling et al., 2011). However, BIA can be considered a limitation because it underestimates BF% and overestimates FFM, particularly in individuals who are categorized as obese (Coppini et al., 2005). Additionally, HRV parameters mostly represent parasympathetic influences on HR, and therefore, we suggested that future studies evaluate the sympathetic activity with other techniques such as microneurography and systolic time intervals for better understanding the ANS activity and its relationship with AS. Moreover, in this study, we did not measure carotid-femoral stiffness, which is the gold standard and a more reliable method for measuring AS (Tanaka et al., 2009).

The strengths of this study included a randomized control design, with strictly controlled supervised exercise sessions and matched caloric expenditure for both training protocols. Our higher intensity protocol could be an attractive option given the time efficiency associated with the design and prescription of exercise training for adult men with sedentary behavior. However, more studies are needed to be specified in different populations (i.e., individuals with obesity and cardiovascular patients) to confirm the effectiveness and safety of the protocols of this study.

## Conclusion

This study demonstrates that both HVMIT and LVHIT protocols improved some HRV variables. However, these improvements were significantly greater in the LVHIT protocol. Moreover, RMSSD, LF, PWV, SBP, VO_2_max, and BF% improved only in the LVHIT protocol following 12 weeks of exercise. Based on these findings, exercise intensity was associated with higher benefits rather than exercise volume. Thus, the higher intensity of exercise training needs to be considered when planning an exercise intervention to improve the ANS activity, AS, cardiorespiratory fitness, and body composition of sedentary adult men. However, further studies are needed to confirm these findings in other populations and further elucidate the mechanisms involved in such adaptations, including the potential association between HRV parameters and PWV after training regimens.

## Data Availability Statement

The raw data supporting the conclusions of this article will be made available by the authors, without undue reservation.

## Ethics Statement

The studies involving human participants were reviewed and approved by the Institutional Review Board of Islamic Azad University (Ethics code: IR-IAU1398-18). The patients/participants provided their written informed consent to participate in this study.

## Author Contributions

MS, MB, AS, DB, AD, and HZ contributed to the conception and design of the study. MS, AD, AS, HZ, KD, SM, and TV contributed to the acquisition, analysis, or interpretation of data. MS, PD-B, DB, BK, and TV drafted the manuscript. All authors critically revised the manuscript, and gave final approval and agreed to be accountable for all aspects of work ensuring integrity and accuracy.

## Conflict of Interest

The authors declare that the research was conducted in the absence of any commercial or financial relationships that could be construed as a potential conflict of interest.

## Publisher’s Note

All claims expressed in this article are solely those of the authors and do not necessarily represent those of their affiliated organizations, or those of the publisher, the editors and the reviewers. Any product that may be evaluated in this article, or claim that may be made by its manufacturer, is not guaranteed or endorsed by the publisher.
